# Successful Point-Of-Care Ultrasound-Guided Treatment of Submassive Pulmonary Embolism

**DOI:** 10.5811/cpcem.2017.7.34504

**Published:** 2017-10-06

**Authors:** Samantha J. Myers, Thomas E. Kelly, Jeffrey R. Stowell

**Affiliations:** Maricopa Integrated Health System, Department of Emergency Medicine, Phoenix, Arizona

## Abstract

Pulmonary embolism is associated with significant mortality and impaired long-term functional outcomes. Timely identification and treatment is crucial for successful management. Unfortunately, prompt diagnosis can be challenging in patients without overt signs of cardiovascular compromise. Point-of-care cardiac ultrasound (POCCUS) can be used to identify signs of acute pulmonary embolism, risk stratify patients for adverse outcomes and assess response to therapy. This report describes a patient with submassive pulmonary embolism and evidence of acute right ventricular strain on POCCUS successfully treated with thrombolytic therapy. The dynamic changes observed on point-of-care ultrasound are presented.

## INTRODUCTION

Pulmonary embolism (PE) is a common and significant condition affecting one to two out of every 1,000 patients presenting to the emergency department (ED).^1^ PE morbidity and mortality is the result of acute right ventricular (RV) outflow obstruction, which leads to compromised RV function and cardiovascular collapse. Mortality varies by clot burden, reaching 65% in massive PE.^1^–^2^ Timely identification and treatment is crucial and has been associated with lower mortality rates and improved long-term functional outcomes.^3^

Unfortunately, prompt and accurate bedside diagnosis can be challenging. Only 5% of acute PE patients may present with observable signs of shock.^4^ The remaining patients, with non-massive PE, are at increased risk of delayed diagnosis. Further, patients with latent RV dysfunction may still develop rapid cardiopulmonary compromise, with significant risk of mortality within the first hour of presentation.^5^,^6^ Rapid identification of RV compromise in these patients is critical to guide treatment, decision-making and disposition planning.

While a variety of diagnostic modalities for identifying RV dysfunction exist, comprehensive transthoracic echocardiography (TTE) is the standard. Signs of RV strain, including RV dilation and hypokinesis, may be readily identified on TTE in up to 30% of normotensive patients.^7^–^11^ Although evaluation of RV function aids in the management of acute PE, comprehensive TTE is not universally available. Alternatively, point-of-care cardiac ultrasound (POCCUS) is an available tool that can be used by emergency physicians (EP) at the bedside to identify signs of acute PE, risk stratify patients for adverse outcomes due to RV dysfunction and assess response to therapy.^6^ We report a case of submassive PE successfully managed with thrombolytic therapy guided by EP-performed POCCUS in which rapid improvement of RV dysfunction was observed.

## CASE REPORT

A 33-year-old female with a history of mild intermittent asthma was sent from a mental and behavioral healthcare facility for evaluation of acute dyspnea. Her dyspnea began three hours prior to arrival and was associated with chest tightness, which she described as similar to previous asthma exacerbations. In addition, her medical history included hypertension, generalized anxiety disorder, bipolar disorder, and polysubstance abuse. She denied hemoptysis, lower extremity swelling, history of prior deep vein thrombosis or PE, estrogen use, current or prior malignancy, recent surgery or trauma.

**Image 1:**
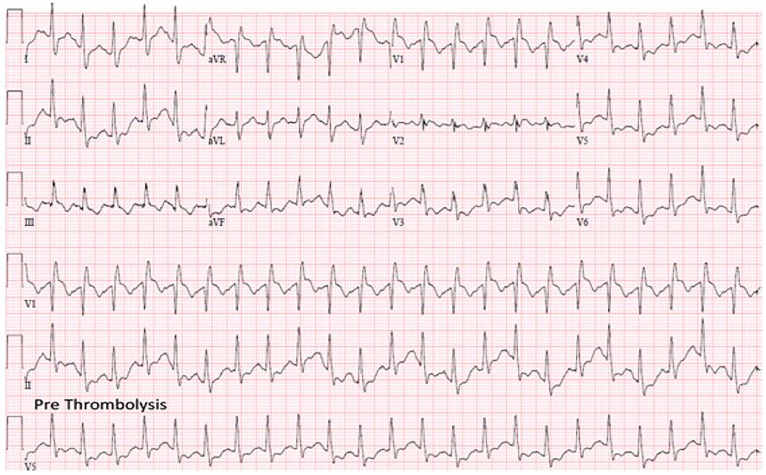
Pre-thrombolysis electrocardiogram demonstrating right heart strain pattern with a right bundle branch block.

Triage vital signs were temperature 36.5°C, blood pressure 106/58 mmHg, heart rate 120 beats per minute, respiratory rate 32 breaths per minute, and oxygen saturation 92% on room air. She appeared anxious and unable to sit still on the gurney. Her lungs were clear to auscultation with symmetric air movement bilaterally without wheezing. Her exam was otherwise normal. Her electrocardiogram (ECG) demonstrated sinus tachycardia with right bundle branch block (RBBB) (Image 1). Portable chest radiography demonstrated no cardiomegaly, pulmonary edema, pleural effusion, or pneumonia.

On initial impression, the diagnosis of pulmonary embolism was considered. The patient was risk stratified with a Wells Criteria for Pulmonary Embolism score of one and a half, for tachycardia, and a Pulmonary Embolism Rule-out Criteria (PERC) score of two, for tachycardia and hypoxemia.^12^, ^13^ With a low-risk Wells score and a positive PERC score, a D-dimer was obtained in addition to cardiac biomarkers and lactic acid. She was treated initially with intravenous (IV) fluids and aspirin 324mg, but she continued to be restless, refusing oxygen and unable to sit comfortably.

**Image 2:**
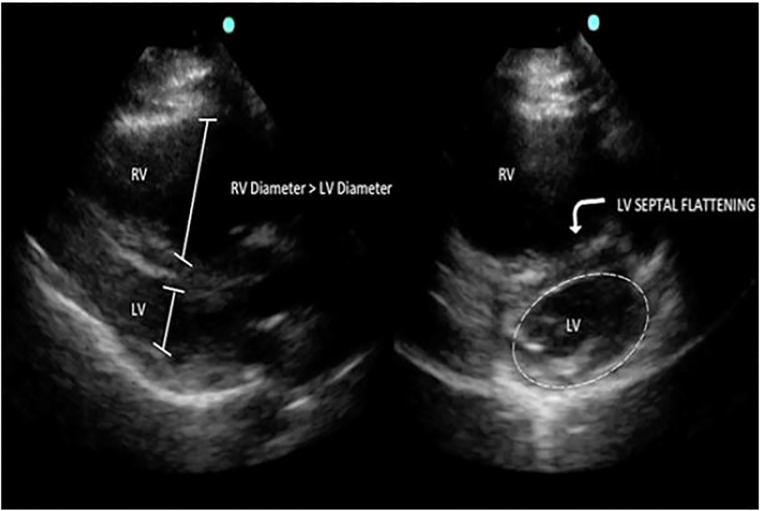
Point-of-care cardiac ultrasound performed pre-thrombolysis. Parasternal long axis view (left) and parasternal short axis view (right) demonstrating right heart strain. *LV*, left ventricle; *RV*, right ventricle.

Laboratory results were significant for elevated D-dimer of 2524 ng/mL (normal <230 ng/mL), B-type natriuretic peptide (BNP) 7295 pg/mL, troponin I 0.117 ng/mL, and lactic acid 5.4 mmol/L. POCCUS demonstrated signs of RV strain with a dilated RV on the parasternal long axis (PLAX) (Image 2) and septal flattening with RV dilation on the parasternal short axis (PSAX) view (Image 2). Computed tomography pulmonary angiography (CTPA) confirmed extensive thrombus within the distal right main pulmonary artery extending into the segmental and subsegmental branches, as well as the segmental and subsegmental branches supplying the left upper and lower lobes.

**CPC-EM Capsule**

What do we already know about this clinical entity?

*Point-of-care cardiac ultrasound (POCCUS) is a skill that emergency physicians can perform rapidly at the bedside to assess for right ventricular (RV) strain in submassive* pulmonary embolism *(PE) to identify patients at risk of hemodynamic decompensation.*

What makes this presentation of disease reportable?

We present POCCUS images obtained before and after successful thrombolysis of PE. Resolution of RV dysfunction was observed.

What is the major learning point?

Resolution of RV dysfunction was visualized only one hour after thrombolytic infusion was initiated. POCCUS applications for evaluating RV strain are reviewed.

How might this improve emergency medicine practice?

Protocols using POCCUS to guide systemic thrombolytic therapy with a goal of preventing cardiovascular collapse may pose a safe option for managing patients with submassive PE.

**Image 3:**
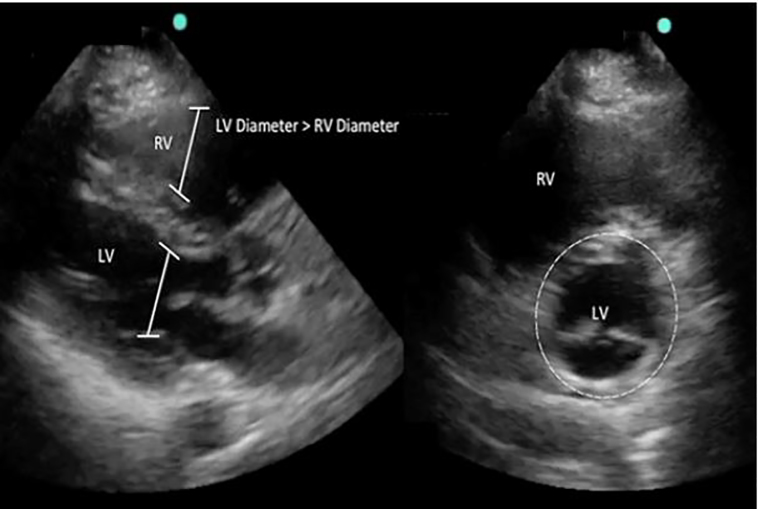
Point-of-care cardiac ultrasound post-thrombolysis. Parasternal long axis view (left) and parasternal short axis view (right) demonstrating resolution of right heart strain. *LV*, left ventricle; *RV*, right ventricle.

**Image 4:**
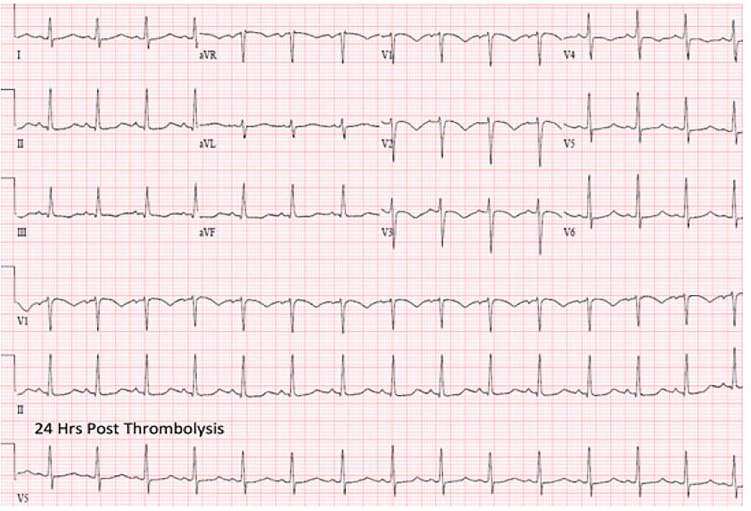
Post-thrombolysis electrocardiogram demonstrating resolution of right heart strain pattern.

Treatment with therapeutic heparin was initiated. Despite this, the patient remained dyspneic, uncomfortable and anxious with no improvement in vital signs. The risks and benefits of thrombolytic therapy, compared to observation, were discussed in depth with the patient. She elected for thrombolytic therapy and was treated with alteplase 100 mg infused over two hours. Repeat POCCUS performed one hour after thrombolytic administration demonstrated significant improvement of RV strain (Image 3), and repeat ECG at 24 hours showed resolution of the RBBB (Image 4). Complications included only minor bleeding from her nose, mouth, and peripheral IV sites. She was admitted to the medical intensive care unit and discharged to the psychiatric facility on hospital day three on oral anticoagulation.

## DISCUSSION

This case highlights important concepts in the management of acute PE, particularly the utility of EP-performed POCCUS for identifying RV dysfunction and treatment response. Real-time differentiation of normotensive patients with acute PE at risk for deterioration is often difficult. Patients may maintain normal vital signs until the moment of deterioration, elevation in troponin or BNP due to myocardial necrosis may be delayed for hours, and comprehensive TTE may not be available. EP-performed POCCUS is an important risk stratification tool in acute non-massive pulmonary embolism and has been established as highly accurate for identifying the presence of RV dysfunction.^14^,^15^ Further, Taylor et al. demonstrated that RV dysfunction on POCCUS was the most important predictive factor for in-hospital adverse events, including death, shock, respiratory failure, and transfer to a higher level of care, with an odds ratio of 9.2 and positive likelihood ratio (LR) of 4.0.^6^

The simplest POCCUS method to identify RV strain is through a visual estimation of the RV to left ventricular (LV) diameter ratio (Image 2). A normal ratio is <1:1, which can be evaluated in the apical four-chamber (A4C) or PLAX views.^16^ In these views, each ventricle’s cross-sectional diastolic diameter can be measured for comparison, and RV dilation can be identified. Additionally, RV dilation has been demonstrated to be 98% specific for the diagnosis of PE, with a positive LR of 29.^14^

The diagnosis of RV strain can be further established by the presence of flattening of the interventricular septum or bowing of the septum into the LV. The PSAX view can aid in identifying these abnormalities. The “D” sign refers to a flattening of the interventricular septum in the PSAX view resulting in a “D”-shaped ventricle, compared to the normal “O” shape, indicating elevated RV pressures (Image 2 right). Paradoxical septal wall motion compromises LV output, contributes to hypotension, and may be a sign of impending hemodynamic collapse. POCCUS using the above methods has been demonstrated to have a sensitivity of 100% and specificity of 99% for RV dysfunction, when compared to comprehensive TTE.^15^

In the setting of increased pulmonary artery pressures, the RV free wall will also become hypokinetic, as it is too weak to contract against the sudden increase in filling pressures. The RV apex may continue to beat hyperdynamically in an attempt to maintain adequate cardiac output. This is best seen on an A4C view at the apex of the RV, where the hyperdynamic apex meets the hypokinetic and dilated RV free wall. This RV hypokinesis with apical sparing is referred to as “McConnell’s sign” and is 94% specific for acute PE.^10^

Tricuspid annular plane systolic excursion (TAPSE) measurement is another useful application for identifying RV strain in acute PE. TAPSE is measured as the displacement of the tricuspid annulus from end-diastole to end-systole. TAPSE measurement is obtained with the A4C view using motion mode (M-mode). The M-mode cursor is placed from the apex of the heart through the lateral tricuspid annulus allowing for visualization and measurement of annulus movement over time. Displacement of the annulus of <16mm is consistent with RV strain. Kopecna et al. demonstrated TAPSE measurement to be the least user-dependent and most reproducible echocardiographic finding of RV dysfunction in normotensive patients with PE.^17^

The presence of right heart thrombus in the setting of PE is associated with worsened outcomes and may be identified with POCCUS. In an analysis of 1,113 patients with acute PE, the presence of RV thrombus on baseline echocardiography was associated with nearly twice the mortality rate at 14 days (21% vs. 11%) and three months (29% vs. 16%) as compared to those without.^18^ Further, the use of thrombolytic therapy in patients with RV thrombus in acute PE has been shown to reduce mortality as compared to heparin alone (p < 0.05).^18^,^19^

The acute echocardiographic abnormalities associated with PE resolve over time with treatment and recovery.^20^ Previous studies have demonstrated that with traditional heparin-based therapy, signs of RV dysfunction on TTE may persist for weeks to months after initiation of treatment.^8^, ^16^ Alternatively, abnormalities may resolve in hours after thrombolytic therapy.^21^ In our patient, improvement in RV dysfunction was identified within 60 minutes after completion of thrombolytic therapy (Image 3). While long-term benefit in functional outcomes with systemic thrombolytic therapy in normotensive patients with acute PE has not been consistently demonstrated, serial POCCUS application may be useful to identify early signs of successful treatment.

## CONCLUSION

POCCUS is a skill that EPs can perform rapidly at the bedside to assess for RV strain in submassive PE. POCCUS applications for evaluating RV strain include identification of RV dilation and hypokinesis, impaired septal wall motion, McConnell’s sign, and TAPSE measurement. This case also highlights the utility of POCCUS in identifying resolution of RV dysfunction after the administration of systemic thrombolytic therapy. Protocols incorporating POCCUS to initiate and guide therapy with a goal of preventing cardiovascular collapse in high-risk patients may pose a safe option.

## References

[1] Cho JH, Kutti Sridharan G, Kim SH (2014). Right ventricular dysfunction as an echocardiographic prognostic factor in hemodynamically stable patients with acute pulmonary embolism: A meta-analysis. BMC Cardiovasc Disord.

[2] Konstantinides SV, Torbicki A, Agnelli G (2014). 2014 ESC Guidelines on the diagnosis and management of acute pulmonary embolism. Eur Heart J.

[3] Jelinek GA, Ingarfield SL, Mountain D (2009). Emergency department diagnosis of pulmonary embolism is associated with significantly reduced mortality: A linked data population study. Emerg Med Australas.

[4] Fengler BT, Brady WJ (2009). Fibrinolytic therapy in pulmonary embolism: an evidence-based treatment algorithm. Am J Emerg Med.

[5] Wood KE (2002). Major pulmonary embolism: review of a pathophysiologic approach to the golden hour of hemodynamically significant pulmonary embolism. Chest.

[6] Taylor RA, Davis J, Liu R (2013). Point-of-care focused cardiac ultrasound for prediction of pulmonary embolism adverse outcomes. J Emerg Med.

[7] Kucher N, Rossi E, De Rosa M (2005). Prognostic role of echocardiography among patients with acute pulmonary embolism and a systolic arterial pressure of 90 mm Hg or higher. Arch Intern Med.

[8] Jardin F, Dubourg O, Guéret P (1987). Quantitative two-dimensional echocardiography in massive pulmonary embolism: emphasis on ventricular interdependence and leftward septal displacement. J Am Coll Cardiol.

[9] Okubo S, Miyatake K, Nagata S (1989). Role of echocardiography in acute pulmonary embolism. Jpn Heart J.

[10] McConnell MV, Solomon SD, Rayan ME (1996). Regional right ventricular dysfunction detected by echocardiography in acute pulmonary embolism. Am J Cardiol.

[11] Grifoni S, Olivotto I, Cecchini P (2000). Short-term clinical outcome of patients with acute pulmonary embolism, normal blood pressure, and echocardiographic right ventricular dysfunction. Circulation.

[12] Wolf SJ, McCubbin TR, Feldhaus KM (2004). Prospective validation of Wells Criteria in the evaluation of patients with suspected pulmonary embolism. Ann Emerg Med.

[13] Kline JA, Courtney DM, Kabrhel C (2008). Prospective multicenter evaluation of the pulmonary embolism rule-out criteria. J Thromb Haemost.

[14] Dresden S, Mitchell P, Rahimi L (2014). Right ventricular dilatation on bedside echocardiography performed by emergency physicians aids in the diagnosis of pulmonary embolism. Ann Emerg Med.

[15] Weekes AJ, Thacker G, Troha D (2016). Diagnostic accuracy of right ventricular dysfunction markers in normotensive emergency department patients with acute pulmonary embolism. Ann Emerg Med.

[16] Mansencal N, Joseph T, Vieillard-Baron A (2003). Comparison of different echocardiographic indexes secondary to right ventricular obstruction in acute pulmonary embolism. Am J Cardiol.

[17] Kopecna D, Briongos S, Castillo H (2014). Interobserver reliability of echocardiography for prognostication of normotensive patients with pulmonary embolism. Cardiovasc Ultrasound.

[18] Torbicki A, Galié N, Covezzoli A (2003). Right heart thrombi in pulmonary embolism: results from the International Cooperative Pulmonary Embolism Registry. J Am Coll Cardiol.

[19] Rose PS, Punjabi NM, Pearse DB (2002). Treatment of right heart thromboemboli. Chest.

[20] Come PC (1992). Echocardiographic evaluation of pulmonary embolism and its response to therapeutic interventions. Chest.

[21] Come PC, Kim D, Parker JA (1987). Early reversal of right ventricular dysfunction in patients with acute pulmonary embolism after treatment with intravenous tissue plasminogen activator. J Am Coll Cardiol.

